# Tislelizumab plus chemotherapy versus pembrolizumab plus chemotherapy for the first-line treatment of advanced non-small cell lung cancer: systematic review and indirect comparison of randomized trials

**DOI:** 10.3389/fphar.2023.1172969

**Published:** 2023-06-20

**Authors:** Yimeng Guo, Junting Jia, Zhiying Hao, Jing Yang

**Affiliations:** Department of Pharmacy, Shanxi Province Cancer Hospital/ Shanxi Hospital Affiliated to Cancer Hospital, Chinese Academy of Medical Sciences/Cancer Hospital Affiliated to Shanxi Medical University, Taiyuan, Shanxi Province, China

**Keywords:** non-small cell lung cancer, programmed cell death 1 receptor, immunotherapy, tislelizumab, pembrolizumab

## Abstract

**Purpose:** Pembrolizumab and tislelizumab have demonstrated significant clinical benefits in first-line treatment for advanced NSCLC. However, no head-to-head clinical trial has ever compared the optimal choice. Therefore, we conducted an indirect comparison to explore the optimal choice for advanced NSCLC combined with chemotherapy.

**Methods:** We conducted a systematic review of randomized trials; the clinical outcomes included overall survival (OS), progression-free survival (PFS), objective response rate (ORR), and adverse events (AEs). Indirect comparisons between tislelizumab and pembrolizumab were conducted with the Bucher method.

**Results:** Data were abstracted from 6 randomized trials involving more than 2,000 participants. Direct meta-analysis showed that both treatment regimens improved clinical outcomes compared with chemotherapy alone (PFS: hazard ratio (HR)_tis+chemo/chemo_ 0.55, 95% CI 0.45–0.67; HR_pem+chemo/chemo_ 0.53, 95% CI 0.47–0.60; ORR: relative risk (RR)_tis+chemo/chemo_ 1.50, 95% CI 1.32–1.71; RR_pem+chemo/chemo_ 1.89, 95% CI 1.44–2.48). Regarding safety outcomes, tislelizumab and pembrolizumab have a higher risk in the incidence of grade 3 or higher AEs (RR_tis+chemo/chemo_ 1.12, 95% CI 1.03–1.21; RR_pem+chemo/chemo_ 1.13, 95% CI 1.03–1.24). The indirect comparison showed that there was no significant difference between tislelizumab plus chemotherapy and pembrolizumab plus chemotherapy in terms of PFS (HR: 1.04, 95% CI 0.82–1.31), ORR (RR: 0.79, 95% CI 0.59–1.07), the incidence of grade 3 or higher AEs (RR 0.99, 95% CI 0.87–1.12), and AEs leading to death (RR 0.70, 95% CI 0.23–2.09). In progression-free survival subgroup analysis, the results demonstrate no significant differences in PFS by PD-L1 TPS expression level, age, liver metastasis status, and smoking status between tislelizumab plus chemotherapy and pembrolizumab plus chemotherapy.

**Conclusion:** The efficacy and safety of tislelizumab combination chemotherapy were not substantially different from pembrolizumab combination chemotherapy.

## 1 Introduction

In recent years, programmed cell death protein-1 (PD-1)/programmed death-ligand 1 (PD-L1) inhibitors have drawn much attention in the fields of tumor therapy (Han et al, 2020). Especially in the treatment of advanced non-small cell lung cancer (NSCLC), many PD-1/PD-L1 inhibitors have demonstrated satisfactory efficacy and safety.

According to the European Society for Medical Oncology (ESMO), in metastatic NSCLC (mNSCLC), the treatment approach varies between oncogene-addicted and non-oncogene-addicted NSCLC. It is recommended that patients are assessed using the Eastern Cooperative Oncology Group (ECOG) Performance Status (PS) before starting any treatment regimen. For advanced oncogene-addicted mNSCLC, using agents such as osimertinib, gefitinib, and erlotinib in first-line therapy is recommended. For non-oncogene addicted mNSCLC, therapy is determined by ECOG PS and PD-L1 expression levels. Monotherapy immune check-point inhibitor (ICI) (e.g., pembrolizumab) is the standard treatment for patients with PS 0–1, tumour PD-L1≥50% and without contraindication for ICI. A combination of platinum-based chemotherapy plus PD-1/PD-L1 inhibitor (e.g., pembrolizumab, atezolizumab) is the most common treatment approach for patients with PS 0–1, regardless of tumour PD-L1 status and without contraindication for ICI. For patients with PS 0–2 and who are contraindicated for immunotherapy, platinum-based chemotherapy doublets are first-line therapies based on histological subtype and organ function. For patients with PS of 2, platinum-based doublets should be considered and single agent chemotherapy is an alternative. Patients with PS 3–4 should be offered the best supportive care. (Postmus et al, 2017).

Pembrolizumab was the first PD-1 inhibitor approved by the FDA (Food and Drug Administration) and the first approved for treating previously untreated metastatic NSCLC combined with chemotherapy. In two phase III trials of KEYNOTE-189 and KEYNOTE-407, pembrolizumab plus platinum-based chemotherapy improved efficacy compared with platinum-based chemotherapy alone in both previously untreated metastatic non-squamous NSCLC (Rodríguez-Abreu et al, 2021) and squamous NSCLC (Paz-Ares et al, 2020).

Tislelizumab is a humanized immunoglobulin G4 (IgG4)-variant monoclonal antibody blocking PD-1 that has been approved by the National Medical Products Administration (NMPA) for the first-line treatment of advanced NSCLC in combination with chemotherapy (Lee and Keam, 2020). In RATIONALE 304, tislelizumab plus chemotherapy significantly prolonged progression-free survival (PFS) compared with chemotherapy alone in patients with locally advanced or metastatic non-squamous NSCLC (Lu et al, 2021). Other studies showed that tislelizumab could provide longer quality-adjusted life-years (QALYs)than docetaxel and nivolumab, with a lower price (Zhou et al, 2022; Zhou et al, 2023).

With multiple approved treatments available, it is important to identify the differences in survival and safety outcomes between them and to balance the cost of care for clinical decision-making. Although pembrolizumab and tislelizumab have demonstrated significant clinical benefits in the first-line treatment of advanced NSCLC, there has never been a head-to-head clinical trial comparing the best choice. To address this problem, we evaluated the efficacy of two forms of combined therapy, tislelizumab plus chemotherapy, versus pembrolizumab plus chemotherapy, for the first-line treatment of patients with advanced NSCLC using indirect comparison.

Currently, there are several choices of indirect comparisons, such as network meta-analysis, the Bucher method, matching-adjusted indirect comparison (MAIC), or reconstruction of individual patient data (IPD). MAIC has poor precision when the sample size is small, and the Bucher method is only suitable for simple indirect comparison (Bucher et al, 1997). Researchers must carefully assess the data to choose an appropriate method for pooling effect sizes.

## 2 Methods

### 2.1 Search strategy

We conducted our review following the PRISMA 2020. A systematic search was conducted through PubMed, Embase, Web of Science, and Cochrane Library databases to select randomized controlled trials that compared tislelizumab plus chemotherapy or pembrolizumab plus chemotherapy with chemotherapy for first-line treatment of advanced NSCLC before 11 November 2022. Keywords contain “NSCLC,” “non-small-cell lung cancer,” “non-small cell lung cancer,” “tislelizumab,” and “pembrolizumab.” Studies were restricted to “randomized controlled trial (RCT)" or “clinical trial."

### 2.2 Selection criteria

The inclusion criteria were as follow.

(I) Population: All patients were histologically or cytologically diagnosed with locally advanced (stage IIIB) or metastatic (stage IV) NSCLC;
(II) Interventions: tislelizumab or pembrolizumab plus chemotherapy as first-line treatment;
(III) Controls: chemotherapy alone as the first-line treatment;
(IV) Outcomes: measurements of efficacy and safety;
(V) Study design: randomized phase 2 or 3 clinical trials.

Exclusion criteria.

(I) Insufficient data;
(II) Duplicate reports;
(III) Retrospective study, systematic reviews, meta-analysis, letters or reviews.

### 2.3 Data extraction and quality assessment

Two investigators independently screened articles according to the predetermined eligibility criteria, and discordances were resolved by mutual discussion. The primary information of the enrolled article was extracted as follows: first author, publication years, type of clinical trial, histology type, number of patients, gender, age, treatments, median follow-up, and clinical outcomes. The clinical outcomes included overall survival (OS), progression-free survival (PFS), objective response rate (ORR), and adverse events (AEs). Data from OS and PFS were evaluated using hazard ratios (HRs) with corresponding 95% confidence intervals (CIs), while data from ORR and AEs were evaluated using risk ratio (RR) and its 95% CI. AEs are classified as AEs of grade 3 or higher, and treatment-related AEs lead to death. The incidence rate of the most frequently occurring immune-related AEs includes hypothyroidism, hyperthyroidism, pneumonitis, hepatitis, and severe skin reactions.

The quality of the included studies was assessed with Cochrane risk of bias tools from seven aspects: random sequence generation, allocation concealment, blinding of participants and personnel, blinding of outcome assessment, incomplete outcome data, selective reporting, and other biases. Disagreements were resolved through discussion.

### 2.4 Statistical analysis

All statistical analyses were conducted with RevMan 5.1 software. A traditional meta-analysis was performed to assess the difference in effectiveness between tislelizumab or pembrolizumab plus chemotherapy and chemotherapy alone. OS and PFS were presented with HRs with 95% CIs with the generic inverse variance method. ORR and frequency of AEs were assessed using RRs and 95% CIs. The χ2 test evaluated heterogeneity and we chose the statistic model base on its results. We used a fixed-effect model if *p*-values >0.1; otherwise, the random-effect model was used. The adjusted indirect comparison was calculated using the Bucher method (Bucher et al, 1997). All tests were 2-sided, with an alpha level of 0.05.

## 3 Results

### 3.1 Characteristics of included clinical trials

**FIGURE 1:**
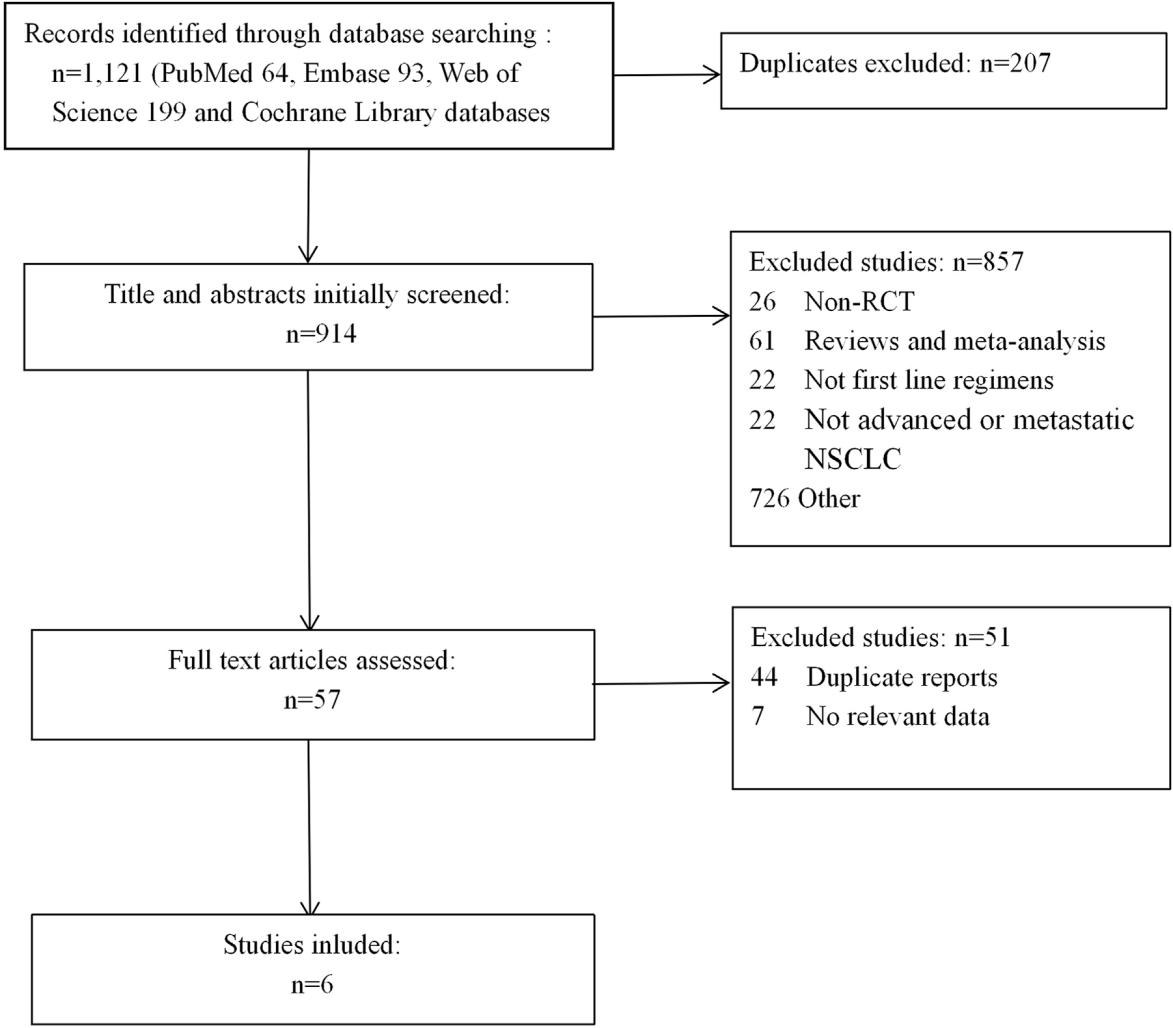
Flow diagram of literature retrieval and selection.

We first identified 1,121 records from online databases (PubMed 64, Embase 93, Web of Science 199, and Cochrane Library databases 765). After excluding duplicates and screening titles and abstracts, 57 studies met our screening criteria. Six studies (Paz-Ares et al, 2020; Awad et al, 2021; Cheng et al, 2021; Lu et al, 2021; Rodríguez-Abreu et al, 2021; Wang et al, 2021) were included (Figure 1), of which Cheng et al was a extension of Paz-Ares et al We decided not to combine the two studies in meta-analysis due to overlap in data. The Cheng et alstudy used only age and smoking status for subgroup analysis of PFS, as relevant data were not available from the Paz-Ares study.

**FIGURE 2:**
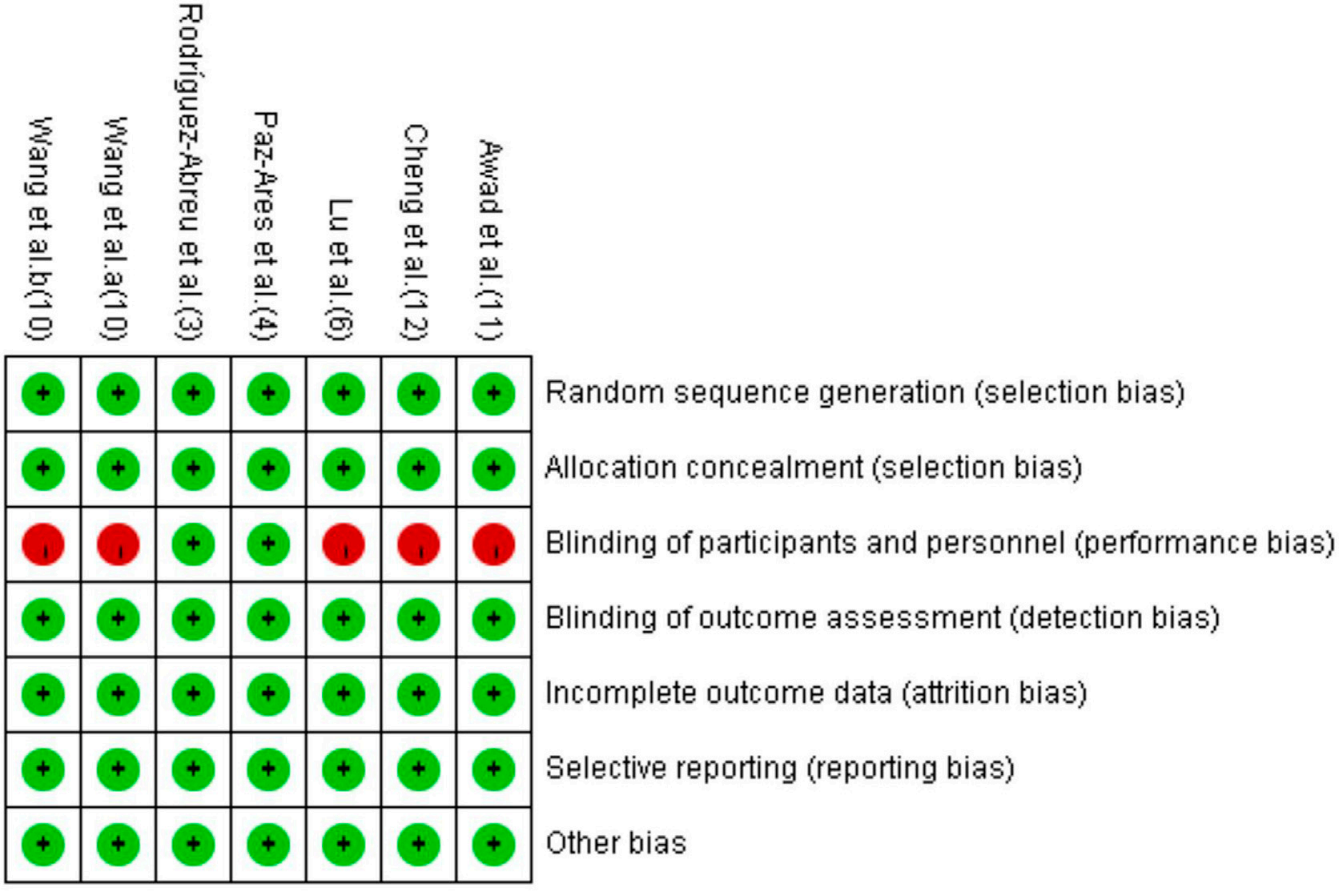
Risk of bias summary for each study.

The detailed information on study characteristics is summarized in Table 1. We summarized the risk of bias results in Figure 2. The high risk of bias was mainly caused by performance bias since four of the included RCTs were open-label studies.

**TABLE 1:** Characteristics of Patients Comparing tislelizumab plus Chemotherapy or Pembrolizumab plus Chemotherapy with Chemotherapy in Included Trials.

Author	Clinical trial type	Histology	Therapeutic regimen	Male (%)		Age (median, years)		No. of patients		Median follow-up (months)	Outcome measures
				Tis/Pem + chemo	Chemo	Tis/Pem + chemo	Chemo	Tis/Pem + chemo	Chemo		
Wang et al (Wang et al, 2021)	Phase III	squamous	Tis + TC vs. TC	89.2	91.7	60	62	120	121	8.6	PFS, ORR, AEs
			Tis + nab-TC vs. TC	94.1		63		119			
Lu et al (Lu et al, 2021)	Phase III	nonsquamous	Tis + PP vs. PP	79.1	83.6	65	65	223	111	9.8	PFS, ORR, AEs
Awad et al (Awad et al, 2021)	Phase II	nonsquamous	Pem + PC vs. PC	37	41	62.5	66	60	63	49.4	PFS, ORR, OS, AEs
Rodríguez-Abreu et al (Rodríguez-Abreu et al, 2021)	Phase III	nonsquamous	Pem + PP vs. PP	62	52.9	65	63.5	410	206	31.0	PFS,ORR, OS, AEs
Paz-Ares et al (Paz-Ares et al, 2020)	Phase III	squamous	Pem + TC/nab-TC vs. TC/nab-TC	79.1	83.6	65	65	278	281	14.3	PFS, ORR, OS, AEs
Cheng et al (Cheng et al, 2021)	Phase III	squamous	Pem + TC/nab-TC vs. TC/nab-TC	95.4	95	63	63	65	60	28.1	PFS

Tis, tislelizumab; nab, nanoparticle albumin-bound; TC, paclitaxel and carboplatin; PP, pemetrexed and platinum; PC, pemetrexed and carboplatin; Pem, pembrolizumab; PFS, progression-free survival; ORR, objective response rate; OS, overall survival; AEs, adverse events.

### 3.2 Direct meta-analysis and indirect meta-analysis

#### 3.2.1 Progression-free survival

**FIGURE 3:**
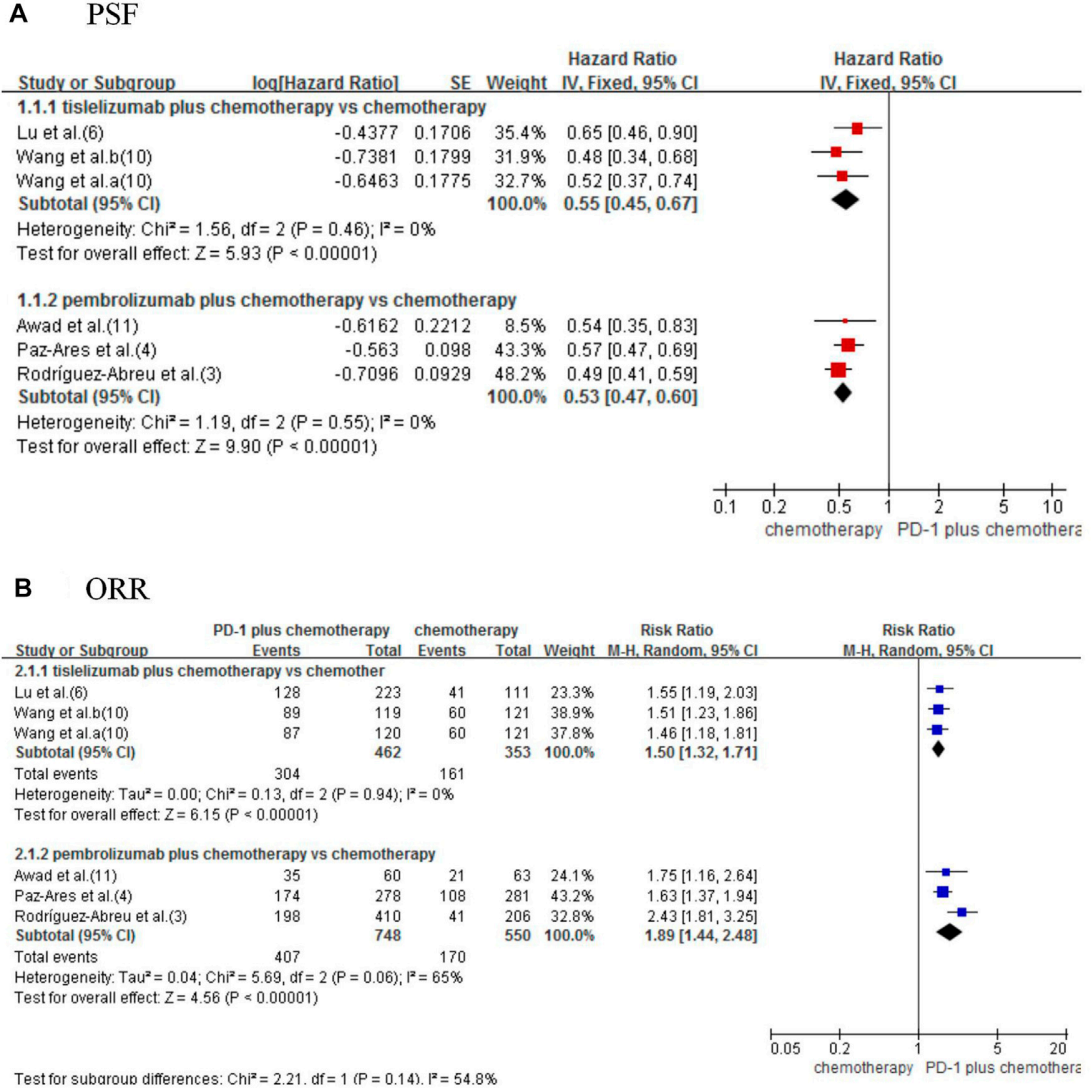
Forest plot for progression-free survival **(A)** and objective response rate **(B)** that compared tislelizumab or pembrolizumab plus chemotherapy with chemotherapy alone in NSCLC patients.

Five studies (Paz-Ares et al, 2020; Awad et al, 2021; Lu et al, 2021; Rodríguez-Abreu et al, 2021; Wang et al, 2021) were included for analysis to estimate the PFS. The result showed that combined therapy significantly improved PFS compared with chemotherapy alone (HR_tis+chemo/chemo_ 0.55, 95% CI 0.45–0.67; *p* < 0.00001; HR_pem+chemo/chemo_ 0.53, 95% CI 0.47–0.60; *p* < 0.00001; Figure 3A).

Using the indirect comparison suggested an insignificant difference between tislelizumab and pembrolizumab (HR _tis+chemo/pem+chemo_: 1.04, 95% CI 0.82–1.31; *p* = 0.77; Table 2).

**TABLE 2:** Indirect Comparison of Tislelizumab Plus Chemotherapy vs. Pembrolizumab Plus Chemotherapy for Advanced NSCLC.

Item	Statistical analysis			
	HR/RR	95%CI		*p*-value
PFS				
Overall	1.04	0.82	1.31	0.77
PD-L1 TPS<1%	1.04	0.72	1.51	0.82
PD-L1 TPS ≥1%	0.98	0.71	1.34	0.89
age <65 years	1.27	0.90	1.80	0.17
age ≥65 years	1.09	0.70	1.69	0.70
Liver metastasis at baseline	0.74	0.38	1.45	0.38
Current or former Smoking	1.11	0.84	1.49	0.46
ORR	0.79	0.59	1.07	0.13
AEs leading to death	0.70	0.23	2.09	0.52
grade 3 or higher AEs	0.99	0.87	1.12	0.85
Hypothyroidism	5.40	0.97	30.01	0.05
Hyperthyroidism	1.65	0.20	13.90	0.64
Pneumonitis	4.76	0.78	28.83	0.09
Severe skin reactions	4.43	0.69	28.38	0.12
Hepatitis	0.12	0.01	1.28	0.08

#### 3.2.2 Progression-free survival subgroup analysis

**FIGURE 4:**
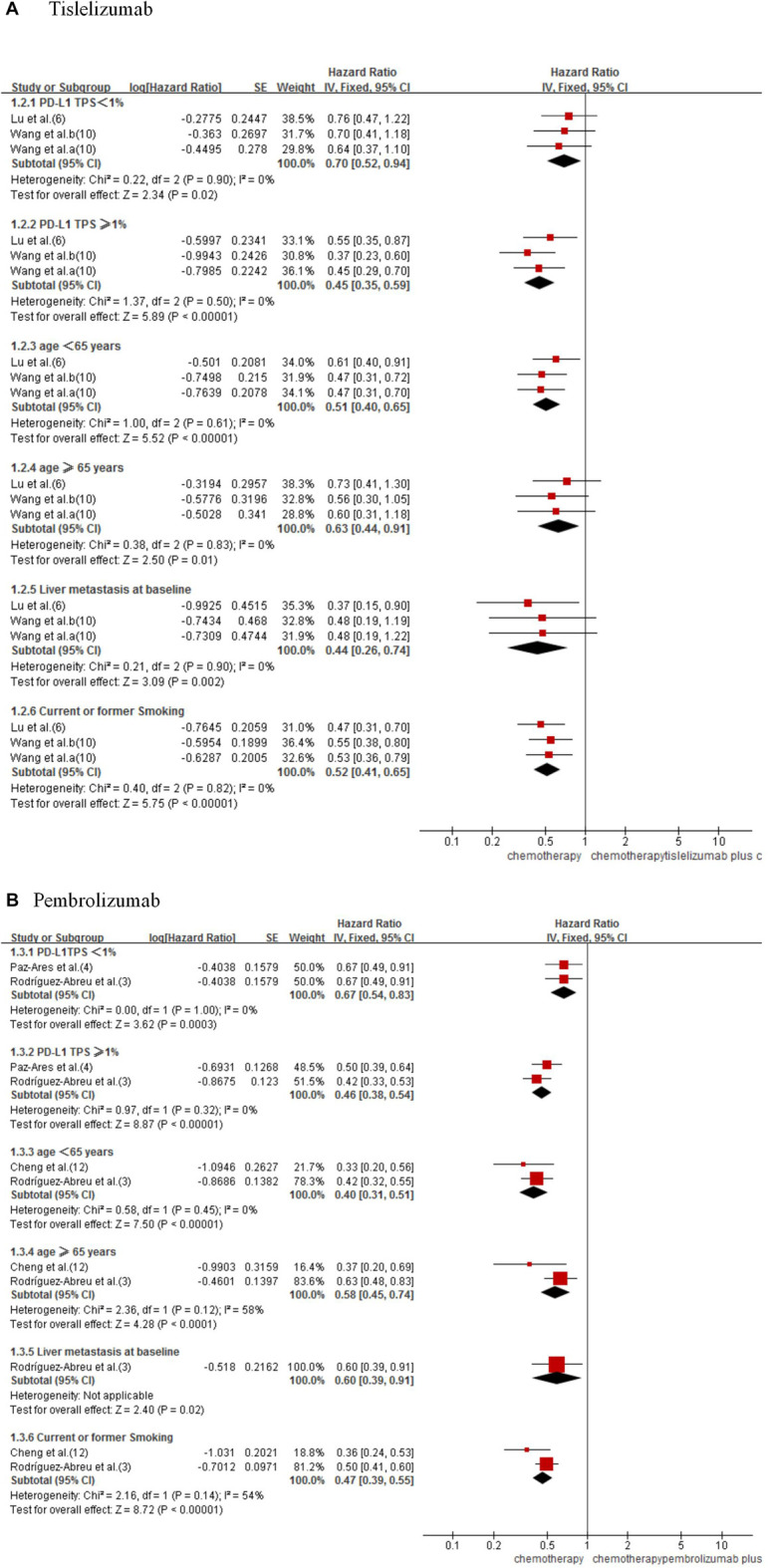
Forest plot for PFS subgroups analysis of tislelizumab **(A)** and pembrolizumab **(B)** plus chemotherapy with chemotherapy alone in NSCLC patients.

In subgroups analysis considering PD-L1 tumor proportion score (TPS) expression level (TPS<1% or TPS≥1%) (3,4,6,10), age (age <65 years or age ≥65 years) (3,6,10,12), liver metastasis status (3,6,10), and smoking status (3,6,10,12), the combined therapy shows better PFS (Figure 4). In indirect comparison, the results failed to demonstrate significant differences in PFS by PD-L1 TPS expression level, age, liver metastasis status, and smoking status between the two forms of combined therapy (Table 2).

#### 3.2.3 Objective response rate

Five studies were included to estimate the ORR (Paz-Ares et al, 2020; Awad et al, 2021; Lu et al, 2021; Rodríguez-Abreu et al, 2021; Wang et al, 2021). Significant improved ORR was observed in combined therapy (RR_tis+chemo/chemo_ 1.50, 95% CI 1.32–1.71; *p* < 0.00001; RR_pem+chemo/chemo_ 1.89, 95% CI 1.44–2.48; *p* < 0.00001; Figure 3B).

Indirect analysis shows no significant difference between the two types of combined therapies (RR 0.79, 95% CI 0.59–1.07; *p* = 0.13; Table 2).

#### 3.2.4 Overall survival

**FIGURE 5:**
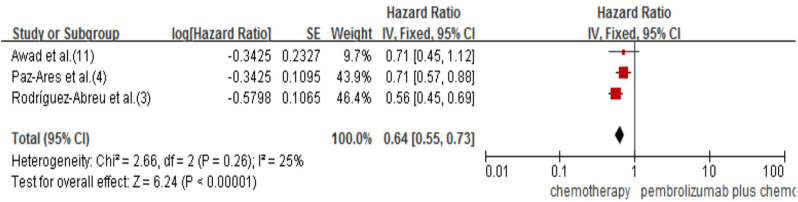
Forest plot for OS that compared pembrolizumab plus chemotherapy with chemotherapy alone in NSCLC patients.

Only three studies reported OS (3,4,11), and they all studied pembrolizumab. The results showed that pembrolizumab plus chemotherapy had a significant effect on improving OS. Compared with the chemotherapy group, it reduced the risk of death by 36% (HR 0.64, 95% CI 0.55–0.73; *p* < 0.00001; Figure 5). For tislelizumab, the median OS was not reached in either of the included studies Lu et al. (2021), Wang et al. (2021); therefore, we could not perform the direct meta-analysis and indirect comparison of OS.

#### 3.2.5 Safety

A total of 5 studies were included in this analysis (Paz-Ares et al, 2020; Awad et al, 2021; Lu et al, 2021; Rodríguez-Abreu et al, 2021; Wang et al, 2021). The results showed that AEs ≥ grade 3 occurred more frequently in the combined therapy group (RR_tis+chemo/chemo_ 1.12, 95% CI 1.03–1.21; *p* = 0.01; RR_pem+chemo/chemo_ 1.13, 95% CI 1.03–1.24; *p* = 0.01). But no significant difference was discovered in AEs leading to death (RR_tis+chemo/chemo_ 0.87, 95% CI 0.33–2.33; *p* = 0.79; RR_pem+chemo/chemo_ 1.26, 95% CI 0.76–2.09; *p* = 0.36).

We found no significant results in the indirect analysis of AEs ≥ grade 3 (RR 0.99, 95% CI 0.87–1.12; *p* = 0.85; Table 2) and AEs leading to death (RR 0.70, 95% CI 0.23–2.09; *p* = 0.52; Table 2) between tislelizumab plus chemotherapy and pembrolizumab plus chemotherapy.

For tislelizumab, when immunotherapy was added to standard chemotherapy, an elevated incidence of hypothyroidism (Lu et al, 2021; Wang et al, 2021) (RR 25.62, 95% CI 5.12–128.31; *p* < 0.0001), hyperthyroidism (Lu et al, 2021; Wang et al, 2021) (RR 7.30, 95% CI 1.36–39.25; *p* = 0.02), pneumonitis (Lu et al, 2021; Wang et al, 2021) (RR 13.45, 95% CI 2.46–73.66; *p* = 0.003) and severe skin reactions (Lu et al, 2021; Wang et al, 2021) (RR 7.70, 95% CI 1.48–40.08; *p* = 0.02) were found, except hepatitis (Lu et al, 2021; Wang et al, 2021) (RR 1.20, 95% CI 0.35–4.12; *p* = 0.77). The incidence of immune-related AEs for pembrolizumab plus chemotherapy showed slightly different results from tislelizumab.

When pembrolizumab was added to standard chemotherapy, an elevated incidence of hypothyroidism (Paz-Ares et al, 2020; Awad et al, 2021; Rodríguez-Abreu et al, 2021) (RR 4.75, 95% CI 2.63–8.58; *p* < 0.00001), hyperthyroidism (Paz-Ares et al, 2020; Awad et al, 2021; Rodríguez-Abreu et al, 2021) (RR 3.98, 95% CI 1.12–14.12; *p* = 0.03), pneumonitis (Paz-Ares et al, 2020; Awad et al, 2021; Rodríguez-Abreu et al, 2021) (RR 2.83, 95% CI 1.56–5.15; *p* = 0.0006) and hepatitis (Paz-Ares et al, 2020; Rodríguez-Abreu et al, 2021) (RR 9.89, 95% CI 1.33–73.54; *p* = 0.03) were found, but not in severe skin reactions (Paz-Ares et al, 2020; Awad et al, 2021; Rodríguez-Abreu et al, 2021) (RR 1.74, 95% CI 0.74–4.09; *p* = 0.20). With indirect meta-analysis, no significant difference was found between the two forms of combined therapies in hypothyroidism (RR 5.39, 95% CI 0.97–30.00; *p* = 0.054; Table 2), hyperthyroidism (RR 1.65, 95% CI 0.20–13.90; *p* = 0.64; Table 2), pneumonitis (RR 4.76 95% CI 0.78–28.83; *p* = 0.09; Table 2), hepatitis (RR 0.12 95% CI 0.01–1.28; *p* = 0.08; Table 2) and severe skin reactions (RR 4.43 95% CI 0.69–28.38; *p* = 0.12; Table 2).

## 4 Discussion

PD-1 is an inhibitory receptor expressed on T cells, while its ligand PD-L1 is overexpressed mainly in various types of cancer. Their binding leads to T-cell exhaustion and reduces the cells’ ability to eliminate neoplastic cells (Wilkins et al, 2021). Inhibiting the interaction between PD-1 and its ligands can enhance the cell-mediated immune response by increasing the activation of T cells, thereby promoting anti-tumor responses and cancer cell apoptosis (Guo et al, 2017). Although PD-1/PD-L1 inhibitors have succeeded in cancer treatment, only a fraction of PD-L1 positive cases may benefit from PD-1/PD-L1 inhibitors monotherapy, while other tumors failed to respond well (Patel and Kurzrock, 2015). Thus, treatment optimization is essential for improving outcomes.

Several studies have shown that chemotherapeutic drugs can increase the antigenicity and immunogenicity of cancer cells, block the immunosuppressive pathways of tumor progression and activate the tumor immune response (Srivastava, 2002; Guertin and Sabatini, 2005; Obeid et al, 2007; Zhang et al, 2008; Ramakrishnan et al, 2010; Pol et al, 2015). Therefore, immunotherapy combined with chemotherapy may benefit a wider range of cancer patients. The results of our direct meta-analysis also support the idea that combination therapy improves clinical outcomes compared to chemotherapy alone and is independent of PD-L1 expression levels.

To our knowledge, our study is the first indirect comparison to evaluate the efficacy and safety differences between tislelizumab and pembrolizumab in first-line treatment for advanced NSCLC. In our indirect comparisons, the OS between tislelizumab and pembrolizumab was not assessed because the median OS was not reported in all studies. Although OS is considered the “gold standard” of effectiveness in oncology drug clinical trials, it may be complicated by in-trial crossover and effective subsequent therapies (Gill et al, 2011). As the surrogate endpoints in clinical trials, PFS and ORR could reduce the impact of these confounders and facilitate the early introduction of new effective therapeutic agents into clinical practice (Hamada et al, 2016; Hamada et al, 2018). In indirect comparisons, we found no significant differences in PFS and ORR between tislelizumab plus chemotherapy and pembrolizumab plus chemotherapy. In addition, the similar efficacy of tislelizumab and pembrolizumab did not vary with PD-L1 expression levels, age, liver metastasis, or smoking status at baseline.

Regarding safety, our study showed that tislelizumab has a similar safety profile to pembrolizumab. The Grade 3 or higher AEs occurred more frequently in the combination therapy group than in the chemotherapy group. The death rate due to AEs was low in combination and chemotherapy groups. These results suggest that introducing PD-1 inhibitors has increased treatment toxicity but is still acceptable. Therefore, adverse events should be closely monitored during the combination treatment to ensure patient safety. It is well known that immune-related AEs can be triggered by PD-1 inhibitors, mainly due to excessive immune activation from PD-1 inhibitors (Baxi et al, 2018). Subgroup analysis showed that both tislelizumab and pembrolizumab were associated with an increased risk of developing immune-related AEs; no differences were seen in hypothyroidism, hyperthyroidism, pneumonitis, hepatitis, and severe skin reactions. These results also illustrated that the safety profiles of these two drugs were similar.

Tislelizumab is a humanized IgG4 anti-PD-1 monoclonal antibody specifically engineered to eliminate FcɣR binding on macrophages, thereby abrogating antibody-dependent cell-mediated phagocytosis (ADCP), ultimately avoiding depletion of T-cells and enhancing its anti-tumor activity (Chen et al, 2019). In addition, recent studies have shown that the binding surface of tislelizumab on PD-1 overlaps largely with that of the PD-L1, and the dissociation rate of tislelizumab from PD-1 is extremely low (Hong et al, 2021). These results indicate that tislelizumab has a higher targeting affinity and efficacy. Our analysis showed that tislelizumab and pembrolizumab were similar in effectiveness and safety, but the treatment cost of tislelizumab was much cheaper in China. The cost of treatment for a single cycle of pembrolizumab is 35,836 RMB in China (4734 EUR), whereas only 2,900 RMB (383 EUR) is needed for tislelizumab. Thus, for the first-line treatment of NSCLC, tislelizumab might be a better choice. Of course, this conclusion will require further validation in large-scale, head-to-head randomized clinical trials.

However, there are some limitations to our study. First, our conclusions rely on indirect comparison rather than head-to-head studies. Network meta-analysis is often used to indirectly compare different treatments, especially when survival outcomes and counts are involved. But considering the simple network in our study, the results of network analysis and the Bucher method are likely similar. Second, we lacked data on OS with tislelizumab, which requires further research. Future analysis may apply the Shiny method instead of common network meta-analysis if follow-up duration is available to pool the results considering the time length. Third, the duration of follow-up of tislelizumab studies was shorter, which may have had some influence on the safety and survival outcomes. Fourth, we only have six studies in analysis, and their grouping method varies, so we could not conduct a subgroup analysis according to the histologic type of lung cancer. Fifth, trials of tislelizumab were conducted on specific ethnic groups, and it is well-recognized that ethnicity will significantly impact clinical outcomes. Finally, the risk of publication bias by funnel plot could not be assessed because less than ten trials were included in the analysis. Thus, the interpretation of the study results needs more caution.

In conclusion, the efficacy and safety of tislelizumab combination chemotherapy were not substantially different from pembrolizumab combination chemotherapy. Tislelizumab may be a good choice for the first-line treatment of NSCLC in clinical practice.

## Data Availability

The raw data supporting the conclusion of this article will be made available by the authors, without undue reservation.

## Abbreviations

NSCLC, Non-small cell lung cancer; ESMO, European Society for Medical Oncology; ECOG, Eastern Cooperative Oncology Group; PS, Performance Status; ICI, immune check-point inhibitor; PFS, Progression-free survival; QALYs, quality-adjusted life-years; HR, Hazard ratio; ORR, Objective response rate; RR, Relative risk; OS, Overall survival; AEs, Adverse events; PD-1, Programmed cell death protein-1; PD-L1, Programmed death-ligand 1; FDA, Food and Drug Administration; IgG4, Immunoglobulin G4; NMPA, National Medical Products Administration; CIs, Confidence intervals; RCT, Randomized controlled trial; Tis, Tislelizumab; Chemo, Chemotherapy; Pem, Pembrolizumab; TPS, Tumor proportion scoreL; ADCP, Antibody-dependent cell-mediated phagocytosis.
